# Supplementary material for: Balancing sensitivity and specificity in distinguishing TCR groups by CDR sequence similarity
Source: BMC Bioinformatics. 2019 May 15;20:241. doi: 10.1186/s12859-019-2864-8 (PMC6521430; doi:10.1186/s12859-019-2864-8)
Supplement: Supplementary file 1 — Classification Results Details. (DOCX 18 kb) [file 12859_2019_2864_MOESM1_ESM.docx]

**Balancing sensitivity and specificity in distinguishing TCR groups by CDR sequence similarity**

Neerja Thakkar and Chris Bailey-Kellogg

**Supplementary Tables**

**Supplementary Table 1. Classification Results Details for Twins Repertoires**

| Twin Pair | Threshold | unidentified % | correct % - total | incorrect % - total | correct% - of identified |
| --- | --- | --- | --- | --- | --- |
| Aα | 0.2 | 0.340 | 0.471 | 0.189 | 0.714 |
| Aα | 0.3 | 0.150 | 0.574 | 0.276 | 0.675 |
| Aα | 0.4 | 0.056 | 0.629 | 0.315 | 0.666 |
| Cα | 0.2 | 0.340 | 0.464 | 0.196 | 0.703 |
| Cα | 0.3 | 0.150 | 0.576 | 0.274 | 0.677 |
| Cα | 0.4 | 0.056 | 0.629 | 0.315 | 0.666 |
| Dα | 0.2 | 0.333 | 0.452 | 0.215 | 0.678 |
| Dα | 0.3 | 0.150 | 0.573 | 0.277 | 0.674 |
| Dα | 0.4 | 0.056 | 0.631 | 0.314 | 0.668 |
| Aβ | 0.2 | 0.595 | 0.273 | 0.131 | 0.675 |
| Aβ | 0.3 | 0.262 | 0.462 | 0.275 | 0.627 |
| Aβ | 0.4 | 0.083 | 0.559 | 0.358 | 0.610 |
| Cβ | 0.2 | 0.595 | 0.260 | 0.144 | 0.643 |
| Cβ | 0.3 | 0.262 | 0.444 | 0.294 | 0.601 |
| Cβ | 0.4 | 0.083 | 0.542 | 0.375 | 0.591 |
| Dβ | 0.2 | 0.595 | 0.253 | 0.152 | 0.624 |
| Dβ | 0.3 | 0.262 | 0.442 | 0.295 | 0.600 |
| Dβ | 0.4 | 0.083 | 0.549 | 0.367 | 0.599 |
| Avg α | 0.2 | 0.337 | 0.462 | 0.200 | 0.698 |
| Avg α | 0.3 | 0.150 | 0.574 | 0.276 | 0.675 |
| Avg α | 0.4 | 0.056 | 0.630 | 0.315 | 0.667 |
| Avg β | 0.2 | 0.595 | 0.262 | 0.143 | 0.647 |
| Avg β | 0.3 | 0.262 | 0.450 | 0.288 | 0.609 |
| Avg β | 0.4 | 0.083 | 0.550 | 0.367 | 0.600 |

**Supplementary Table 2. Classification Results Details for Dash et al. repertoires**

| Category | Threshold | unidentified % | correct % - total | incorrect % - total | correct% - of identified |
| --- | --- | --- | --- | --- | --- |
| Human α | 0.2 | 0.464 | 0.484 | 0.052 | 0.903 |
| Human α | 0.3 | 0.356 | 0.557 | 0.087 | 0.866 |
| Human α | 0.4 | 0.277 | 0.612 | 0.111 | 0.847 |
| Human β | 0.2 | 0.533 | 0.428 | 0.040 | 0.9152 |
| Human β | 0.3 | 0.428 | 0.496 | 0.076 | 0.867 |
| Human β | 0.4 | 0.261 | 0.583 | 0.156 | 0.789 |
| Mice α | 0.2 | 0.224 | 0.472 | 0.304 | 0.608 |
| Mice α | 0.3 | 0.080 | 0.508 | 0.412 | 0.553 |
| Mice α | 0.4 | 0.034 | 0.516 | 0.450 | 0.534 |
| Mice β | 0.2 | 0.313 | 0.531 | 0.156 | 0.773 |
| Mice β | 0.3 | 0.121 | 0.586 | 0.293 | 0.666 |
| Mice β | 0.4 | 0.026 | 0.617 | 0.358 | 0.633 |
